# Prolongated Activated Partial Thromboplastin Time (aPTT) in Pediatric Patients before Surgery—Crying Wolf: Lupus (Anticoagulant) Does Not Always Threaten Children

**DOI:** 10.3390/jcm13051510

**Published:** 2024-03-06

**Authors:** Tiziano Martini, Rita Carlotta Santoro, Laura Banov, Antonella Ierardi, Marzia Leotta, Alessandra Strangio, Johanna Svahn, Angelo Claudio Molinari

**Affiliations:** 1 Immuno-Haematology and Transfusion Medicine, Center for Congenital Bleeding Disorders, Cesena General Hospital, 47521 Cesena, Italy; tiziano.martini@auslromagna.it; 2 Hemostasis and Thrombosis Unit, Azienda Ospedaliero Universitaria Dulbecco, 88100 Catanzaro, Italy; ritacarlottasantoro@gmail.com (R.C.S.); antonellaierardi86@gmail.com (A.I.); marzialeotta@virgilio.it (M.L.); alestr87@gmail.com (A.S.); 3 Thrombosis and Hemostasis Unit, IRCCS Istituto Giannina Gaslini, 16147 Genova, Italy; laurabanov@gaslini.org (L.B.); johannasvahn@gaslini.org (J.S.)

**Keywords:** lupus anticoagulant, antiphospholipid antibody syndrome, pediatric, surgery, prolonged activated partial thromboplastin time

## Abstract

A prolonged preoperatory aPTT in children is often the cause of a delay of scheduled surgeries and the repetition of multiple blood tests, with the consequent wasting of resources and significant discomfort for children and parents. The aim of this review is to analyze the situations in which an isolated prolongation of aPTT is found during preoperative evaluation in children, especially when it is due to the presence of antiphospholipid antibodies, providing the readers with the keys to interpret this situation and the possibility to correctly evaluate the hemorrhagic risk of a patient.

## 1. Introduction

Phospholipids play a crucial role in the coagulation process, and certain coagulation tests are termed “phospholipid dependent” because they rely on the presence of phospholipids to accurately reflect the normal coagulation cascade. This is the case for activated partial thromboplastin time (aPTT), a widely used clot-based assay, sensitive to factor defects in the intrinsic and common pathways of coagulation (factors II, V, VIII, IX, X, XI, and XII, fibrinogen, high-molecular-weight kininogen, and prekallikrein). A reduced availability of phospholipids determines a prolongation of this coagulation time [1]; therefore, the aPTT is prolonged in the presence of circulating antiphospholipid antibodies (APAs), a group of heterogeneous circulating autoantibodies that include lupus anticoagulant (LA), anticardiolipin antibodies (aCLs), and anti-β2-glycoprotein I antibodies (aB2GPIs) [2]. These antibodies are directed against complexes formed by negatively charged phospholipids and surface proteins, and represent the laboratory criteria that define antiphospholipid syndrome (APS), a systemic autoimmune disease characterized by thrombotic or obstetrical events that occur in patients with persistent antiphospholipid antibodies [3]. APS can be associated with autoimmune conditions (mainly systemic lupus erythematosus, SLE) and occasionally with infections, drugs, and malignancies (Table 1); APS can also be found in patients with neither clinical nor laboratory evidence of another definable condition (primary APS) [4].

The pathogenetic role of APAs in determining thrombosis is not yet fully understood; these autoantibodies are directed against a number of plasmatic proteins and surface proteins of endothelial cells and platelets, and are probably able to alter the expression as well as secretion of these molecules, leading to the dysregulation of the balance between procoagulant and anticoagulant pathways [4] (Table 2).

Not all patients with APAs develop APS, as such antibodies have been found in about 5% of the healthy population [5].

The estimated incidence of APS ranges between 1 and 2 cases per 100,000. The prevalence of antiphospholipid antibodies in patients with obstetric morbidity is 6–9%, while in arterial and venous events it is 9–10%. APS tends to be more frequent in the female sex [6].

The epidemiology of APS in childhood remains largely uninvestigated. The International Pediatric APS registry and several pediatric APS cohorts have reported a mean diagnosis age of 10 years, apparently without sex differences [7,8].

In children, there is a high incidence of transient APA positivity during infectious conditions, which usually does not carry an increased risk of thrombosis, even if several thromboembolic events have been described in association with these antibodies [9]. Data about incidence, prevalence, clinical features, and thrombotic risk in APA-positive and APS pediatric patients are limited, showing a lack of robust evidence in this setting in the literature.

## 2. Methodology

**Figure 1:**
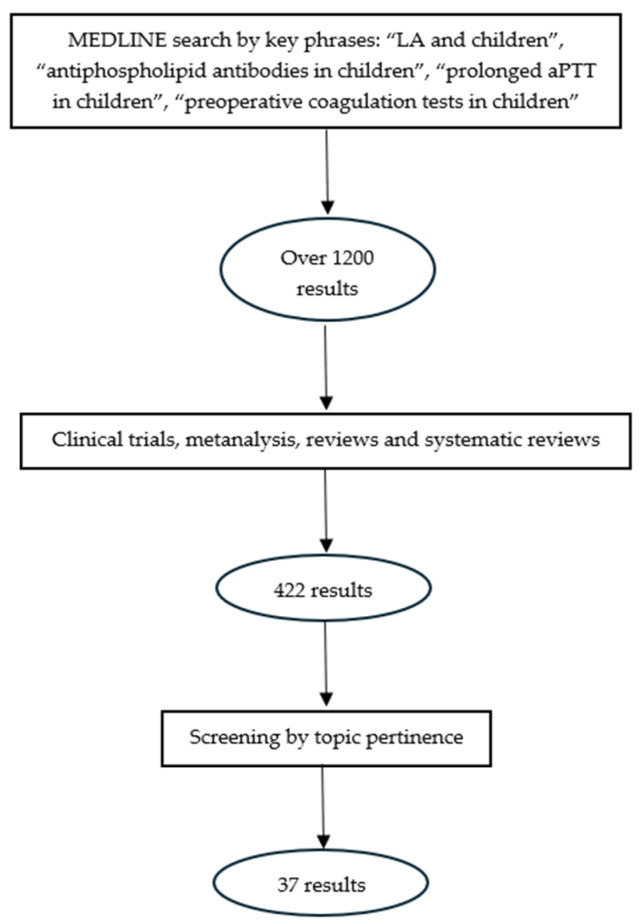
Our methodology of research.

We performed a systematic review of the literature by consulting the MEDLINE database through the PubMed search engine, using the following key phrases: “LA and children”, “antiphospholipid antibodies and children”, “prolonged aPTT in children”, and “preoperative coagulation tests in children”. We obtained over 1200 results, which we filtered by article type, these being clinical trials, meta-analyses, reviews, and systematic reviews; the remaining 422 articles were screened by the pertinence to our topic and 37 works were eventually analyzed. Figure 1 illustrates our methodology of research.

## 3. APAs Associated with Infections: A Peculiar Antibody Pattern?

In the 1990s the APAs associated with autoimmune conditions were considered to be clearly different from those related to infections, being the first thrombogenic ones and needing a “cofactor”, namely β2-glycoprotein I: the two types of antibodies were referred to as “autoimmune” and “infectious” APAs [10]. This distinction, however, has subsequently been found not to be absolute. The main hypothesis that became predominant explained the formation of pathogenic APAs as the consequence of a trigger induced by infections in predisposed subjects [11]. 

Table 3 summarizes the characteristics of APAs in association with viral infections.

Several bacterial and parasite infections can be accompanied by APA positivity (Table 4), generally not related to thromboembolic episodes [12]. In Q fever, aCL IgGs are synthetized during primary infection and incidentally associated with endocarditis development, and are also present in arterial and venous thrombosis in the severe forms [13]. APA-positive Q fever associated with splenic infarction was also observed in children [14].

Another important link between infections and APAs is represented by the catastrophic anti-phospholipid syndrome (CAPS), defined as small-vessel thrombosis in three or more organs in less than one week in the presence of APAs. This severe condition is often triggered by a precipitating event, such as an infection [15,16], and associated with high (50%) mortality, mostly due to cerebral and cardiac thrombosis, infections, and multiorgan failure [17].

Severe acute respiratory syndrome coronavirus 2 (SARS-CoV-2) can also be associated with APAs; a recent large meta-analysis revealed that APAs are present in nearly 50% of the cases, and LA was the most frequently detected antibody; however, no correlation between the APA titers in SARS-CoV-2 and the risk of thrombosis was observed [18].

## 4. Discussion

Transient APA positivity, which disappears with the resolution of an infection, can be associated in children with a number of viral infections, including hepatitis C virus, human immunodeficiency virus (HIV), cytomegalovirus (CMV), varicella zoster, Epstein–Barr virus (EBV), adenovirus, and parvovirus B19. It can sometimes be accompanied by thrombotic events, including renal thrombotic microangiopathies or deep vein thrombosis [19]. 

In pediatric patients, APAs are known to often be associated with recent infections, usually transient (disappearing in parallel with the disappearance of an infection) and generally not clinically significant [20,21,22].

A Turkish study on 165 children affected by recurrent upper respiratory tract infections [23] showed the presence of LA in eight patients (4.8%) and demonstrated the disappearance of APAs soon after the diagnosis (average interval of time of 2 months). Frauenknecht et al. [24] studied APAs in pediatric patients below the age of 14 years with prolonged aPTT during infections: 89.2% of children with prolonged aPTT, elevated C reactive protein levels, and the diagnosis of an infection/fever showed the presence of APAs (almost belonging to all antibody types). 

The risk of venous thromboembolism (VTE) in the first decades of life recognizes two peaks, in neonates and adolescents; the increase in risk in these patients is especially due to acquired factors: venous catheter, infections and sepsis, dehydration, congenital cardiopathies, polycythemia, cancer, polychemotherapy, obesity, rheumatic disease, and the use of oral contraceptive pills [25]. For these reasons, thrombophilia screening is not frequently performed in childhood and adolescence, and it is limited to specific conditions (neonates, children, and adolescents with spontaneous VTE or stroke, or those that are asymptomatic with a very strong familial history) [26]; consequently, the finding of APA positivity is often accidentally due to coagulation exams made for other reasons, especially before surgery. The most typical laboratory finding that originates the search of APAs is a prolonged aPTT, usually performed as a screening coagulation test before any kind of surgery. Tonsillectomy, with or without adenoidectomy, is the most frequent major surgical procedure in a pediatric patient [27], bleeding being the most frequent complication of this procedure, occurring in 1–8% of total cases [27,28]; the normality of coagulation screening tests is an essential requirement to undergo this kind of surgery. Moreover, since mild hemophilia A, B, or C (factor XI deficiency) could not have led to bleedings in the first years of age, surgeons can but diagnose due to a preoperatory prolonged aPTT; anesthesiologists and pediatricians are often worried about possible bleeding complications of tonsillectomy.

A “biological paradox” refers to a prolongation of clotting time, which mimics a bleeding disorder, accompanied by a prothrombotic tendency; however, there is an exception to this paradox, known as the Lupus Anticoagulant-Hypoprothrombinemia Syndrome (LAHS). This is a rare condition, typically found in pediatric patients, where APAs interfere with the activity of factor II. This can result in bleeding episodes in some cases or make patients prone to bleeding after trauma or surgery [29].

Table 5 summarizes the main studies on APA findings in a preoperative setting.

## 5. Suggestions: A (Possible) Way to “Not to Lose the Horizon”

Almost all of the above-mentioned works [9,30,31,32,33,34,35,36,37,38] underline some fundamental aspects, these being as follows:

- The relative low frequency of screening coagulation test abnormalities in the pediatric population investigated prior to surgery.
- A consistent incidence of LA/APAs among children with prolonged aPTT, and its significative relationship with recent infections (especially frequent upper air tract infections in children candidates for adenotonsillectomy).
- The transient character of these antibodies, often disappearing at a second control (performed after a variable interval of time).
- The association between these test abnormalities and the absence of personal and familial histories of bleeding.

Based on what has been summarized above, we believe that pediatricians and surgeons can make a safe decision by carrying out two simple steps.

On the clinical side, it is important to collect the medical history of a patient, using a standardized tool that allows a systematic evaluation of the patient bleeding phenotype; the most accurate bleeding score validated for the pediatric population is the Pediatric Bleeding Questionnaire (PBQ, File S1) [39], developed by Bowman et al., modifying the MCMDM-1 Bleeding Questionnaire for von Willebrand disease [40] by including pediatric-specific bleeding symptoms in the “Other” category: umbilical stump bleeding, cephalohematoma, post-circumcision bleeding, post-venipuncture bleeding, and macroscopic hematuria. This bleeding score has been demonstrated to have high specificity and sensitivity in recognizing von Willebrand disease in children [39], showing a strong correlation with the risk of post-surgery bleeding. A PBQ score > 2 can be considered suspect for a possible bleeding disorder and justifies the evaluation of a patient by a specialized hemostasis center, prior to surgery.

The second step is a simple and fast laboratory method to exclude a silent bleeding disease: the mixing test for aPTT can discriminate if the prolongation of aPTT is due to the deficiency of one or more coagulation factors or to the presence of a circulating anticoagulant directed against one or more of the same factors or against phospholipids; mixing patient plasma with “normal” plasma (normal pool plasma, NPP, obtained from a pool of healthy subjects) will correct the prolongation of aPTT in case of a factor deficiency (because the factor level obtained by adding NPP will determine a clotting time in the range of normality), while the presence of an inhibitor will neutralize both patient factor(s) and NPP factor(s) [1]. This principle justifies the use of mixing tests in LA detection; in the so-called “three steps approach”, after performing a screening assay (namely a PL-dependent assay: aPTT or dRVVT, dilute Russell Viper Venom Time), the coagulation test is repeated in a mixture of patient plasma and NPP: if LA is present in the patient plasma, the amount of coagulation factors provided from the NPP will not correct the prolongation of the clotting time [3]. If the mixing test does not modify aPTT, there is a high probability of the presence of LA, and for the reasons discussed above it is not a contraindication to proceed to surgery; if the mixing test is able to correct aPTT prolongation, the patient must be referred to a specialized hemostasis center prior to surgery.

**Figure 2:**
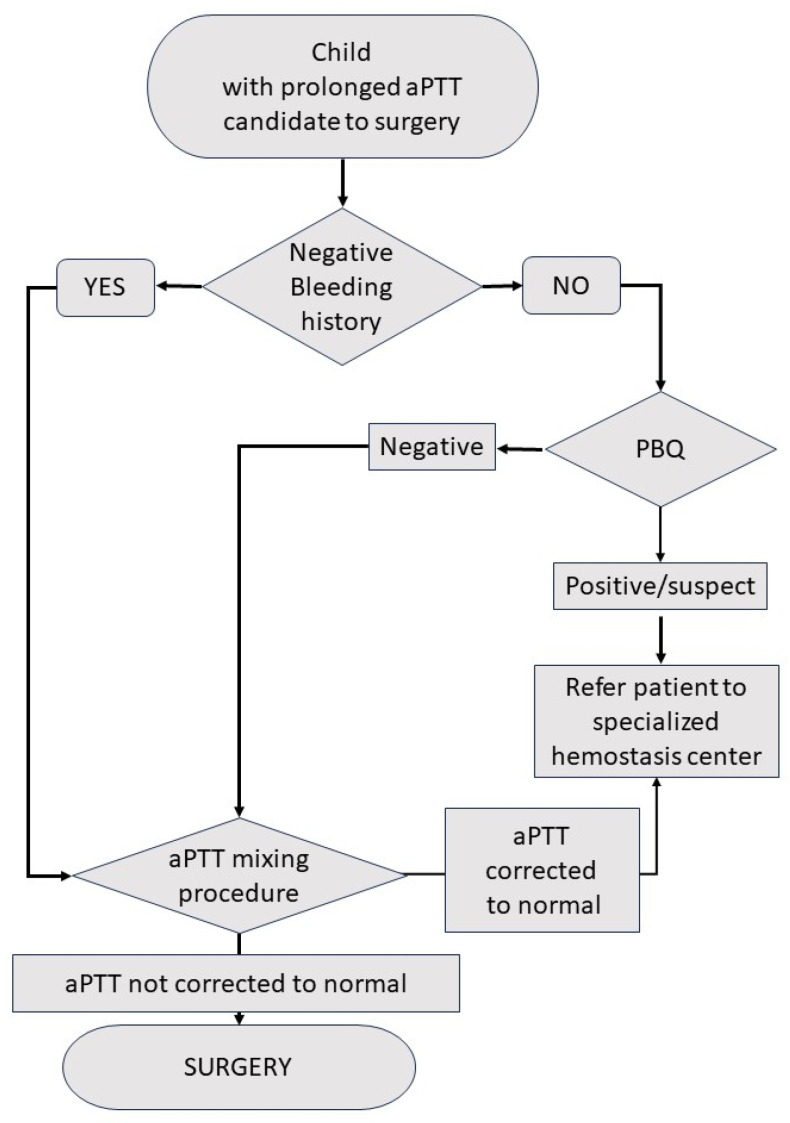
Suggested diagnostic differential approach for an isolated prolonged aPTT in a child during preoperative exams.

The algorithm in Figure 2 represents our diagnostic differential approach.

The strength of our article is its focus on a very common problem that has been little covered in the literature, and in any case little is known about it. Clinicians will therefore benefit greatly from reading our article. On the other hand, the same aspect also represents a weak point, because the substance of the opinion is essentially based on our long clinical experience in the fields of hemostasis and thrombosis.

## 6. Conclusions

The finding of LA positivity in preoperative settings determines several consequences for both clinicians and patients that represent important challenges:

- The need to perform several blood tests with possible difficulties in interpreting their results, which can lead to a diagnostic delay for a condition that is mostly benign and unrelated to increased bleeding risk.
- This diagnostic delay unavoidably provokes a postponement of surgery, with the need for repeated planning of the preoperative evaluation.
- The postponement of surgery and the repetition of blood tests as well as specialist visits lead to significant discomfort for patients and parents, with the need for several hospital visits and an incorrect perception of an unknown bleeding disorder, hesitating in unjustified anxieties and worries.
- Moreover, all these issues ultimately lead to the wasting of resources for families and the health system.

To avoid all this, pediatricians, surgeons, and anesthetists must be able to make the correct choices when faced with a child who must undergo surgery and who presents with a lengthening of aPTT.

This review provides them with simple and effective indications with which to orient themselves while avoiding the wasting of resources and risks for patients.

**Take Home Messages**

- A prolonged and isolated aPTT is a common finding in preoperative testing in children, especially before adenotonsillectomy.
- Pediatricians, surgeons, and anesthetists are often afraid of possible bleeding complications and consequently delay surgery in most cases.
- Correct medical history taking in the clinic and a mixing procedure in the laboratory can help them to easily and quickly make the right decision, reducing the wasting of resources and the inconvenience to families.

## Figures and Tables

**Table 1:** Conditions frequently associated with APAs (adapted from Gómez-Puerta J et al. [4]).

Systemic Autoimmune Diseases	Infections	Malignancies	Drugs
Dermatomyositis Polymyositis Vasculitis Primary Sjogren’s syndrome Rheumatoid arthritis Systemic lupus erythematosus Systemic sclerosis	Bacterial: (syphilis, Lyme disease, tuberculosis, leprosy, infective endocarditis, rheumatic fever, and Klebsiella) Protozoal: (malaria, toxoplasmosis) Viral: (HIV infection, mononucleosis, rubella, parvovirus, hepatitis A, B, and C, and mumps)	Myeloid and lymphatic leukemias Polycythemia vera Myelofibrosis Lymphoproliferative diseases Monoclonal gammapathies Waldenström macroglobulinemia Myeloma Solid tumors (lung, colon, cervix, prostate, liver, kidney, thymus, esophagus, maxilla, ovary, and breast)	Anti-TNFa treatments Oral contraceptives Phenothiazines Ethosuximide Chlorothiazide Quinine Procainamide

**Table 2:** APAs in thrombosis: possible pathogenetic mechanisms (adapted from Gómez-Puerta, J. et al. [4]).

Effects on physiologic coagulation inhibitors Inhibition of B2GPI anticoagulant activity. Inhibition of the protein C pathway. Inhibition of antithrombin activity.
Effects on endothelial cells Enhanced endothelial cell procoagulant activity. Increased expression and activation of tissue factor. Expression of adhesion molecules. Impaired fibrinolysis. Dysregulation of eicosanoids. Decreased endothelial cell prostacyclin production. Increased platelet thromboxane A2 production. Impaired function of endothelial nitric oxide synthase.
Effects on monocytes Expression of tissue factor. Increased oxidative stress.
Effects on platelets Enhanced platelet activation/aggregation.

**Table 3:** Characteristics of APA in association with viral infections (adapted from Ruiz et al. [12]).

Infectious Agent	aCL	aB2GPI	Transient	APS Manifestation
Hepatitis C	IgG	+	+/−	Thrombosis, brain infarction
Epstein–Barr virus	IgG, IgM	+	+	PE, thrombosis
Varicella	IgG, IgM	−	+	PE, thrombosis
Parvovirus B19	IgG	+	+	Thrombosis
Cytomegalovirus	IgG, IgM	+	+	Thrombosis
HIV	IgG, IgM, IgA	+	+/−	Leg ulcer necrosis, PE, VTE, arterial and vein thrombosis, vasculitis, and livedo reticularis
Adenovirus	IgG	+	+	Thrombocytopenia
Herpesvirus-6	IgG	−	+	Cerebral infarct

**Table 4:** Characteristics of APAs in association with bacterial and parasite infections (adapted from Ruiz et al. [12]).

Infectious Agent	Frequency (%)	aCL Isotype
Leprosy	33–67	IgG, IgM, and IgA
Tuberculosis	27–53	IgG, IgM
Bacterial endocarditis	5–44	IgG, IgM
Helicobacter pylori	ND	IgG, IgM
Mycoplasma pneumonia	20–53	IgG, IgM, and IgA
Staphylococcus Aureus	43	IgG, IgM, and IgA
Streptococcus	80	IgG, IgM, and IgA
Streptococcus pyogenes	0–80	IgG, IgM
Malaria	30	IgG, IgM

**Table 5:** Main studies investigating APA finding in preoperative setting.

Study	No. of Patients	Patients Characteristics	Antibody Pattern	Duration of APA Positivity	Thromboembolic Events
Giordano et al. [9]	44	Children with prolonged aPTT (not correct by a mixing test) studied for preoperative coagulation tests or because of abnormal clotting tests, thrombosis, or bleeding	No differences between transiently and persistently positive patients for LA and aCL; IgM anti-B2GPI more frequent in persistent positivity	Of the patients, 25% had APA positivity confirmed after 12 weeks	Four events in persistently positive patients (36%)
Bhasin et al. [30]	792	Children referred to a pediatric hematologist for a prolonged PT and/or aPTT at preoperative testing	LA in 33% of patients, aCL in 1.5%		
Aguirre et al. [31]	38	Children with prolonged aPTT at preoperative testing	LA	LA in 28.5% of patients with persistently prolonged aPTT (median control time of 3 months), namely in 5.2% of total patients	
Samkova et al. [32]	274	Children investigated for preoperative screening	LA in 3% of patients		
Shah et al. [33]	90	Patients with prolonged aPTT (32% of which followed abnormal presurgical evaluations)	LA in 23% of patients, aCL in 12%		
Malbora et al. [34]	68	Children with prolonged aPTT in preoperative screening	LA in 57% of patients	LA positivity disappeared in 64% of patients after 10 weeks on average; 36% had persistent LA positivity	
Male et al. [22]	95	Children with a prior diagnosis of LA; 84% diagnosed incidentally after investigation for preoperative screening	LA	Of the patients, 58% resolved aPTT prolongation after a median period of 1.9 years; 15% had persistent positivity after a median period of 3.1 years	
Jo et al. [35]	66	Children with a prolonged PT and/or aPTT at a routine examination and screening test prior to an invasive procedure	LA in 50% of patients		
Zamudio Penko et al. [36]	1835	Children with prolonged aPTT and/or PT at preoperative screening	LA in 8% of patients		
Song et al. [37]	363	Children with abnormal preoperative coagulation tests prior to minor surgery	LA in 3.9% of patients		
Kang et al. [38]	7114	Children with abnormal preoperative coagulation tests prior to surgery	LA in 14 patients		

## Data Availability

Not applicable.

## Supplementary Materials

The following supporting information can be downloaded at: https://www.mdpi.com/article/10.3390/jcm13051510/s1. File S1. Pediatric Bleeding Questionnaire (PBQ).
