# Supplementary material for: Prolongated Activated Partial Thromboplastin Time (aPTT) in Pediatric Patients before Surgery—Crying Wolf: Lupus (Anticoagulant) Does Not Always Threaten Children
Source: J Clin Med. 2024 Mar 6;13(5):1510. doi: 10.3390/jcm13051510 (PMC10934886; doi:10.3390/jcm13051510)
Supplement: Supplementary file 1 [file jcm-13-01510-s001.zip › jcm-2857178-supplementary.pdf]

Journal of Clinical Medicine

MDPI

Pediatric Bleeding Questionnaire (PBQ) (39)

Subject data

Date

Child's Name Parent's Name

Address Email

Phone Number

Gender Male ☐ Female ☐

Age DOB (dd/mm/yy)

Ethnic Background of: Father Mother

Presenting complaint of bleeding or bruising today? Yes ☐ No ☐

Ever been diagnosed with a bleeding disorder? Yes ☐ No ☐

Diagnosis:

Total # of 1st degree family members □□ # of 1st degree family members studied □□

# of diagnosed 1st degree family members □□ Notes:

ABO Blood Group A ☐ B ☐ AB ☐ O ☐ Rh - ☐ Rh + ☐

Present questionnaire completed by Father ☐ Mother ☐ Subject ☐

Menarche N/A ☐ Yes ☐ No ☐ Age of menarche:

Are you currently taking oral contraceptive pills? Yes ☐ No ☐
Brand Name:

Specify any herbals and/or medications that you have taken in the past 30 days:

Name Dose Route Frequency Duration

____

____

Past Medical History

Temperature (day of blood work)

**Bleeding symptoms**					
**Epistaxis** No □ If Yes,	Trivial □		Significant □		
**AVERAGE PRESENTATION**					
Age of maximum severity	□ 0 - 4 yrs	□ 4 - 8 yrs	□ 8 - 12 yrs	□ 12 - 16 yrs	□ 16 - 20 yrs
Number episodes/year	□ < 1	□ 1 - 5	□ 6 - 12	□ > 12	
Duration of average single episode	□ < 1 minute	□ 1-10 minutes	□ > 10 minutes		
Spontaneous?	Yes □	No □			
Both nostrils?	Yes □	No □			
After drug ingestion (e.g.aspirin)	Yes □	No □			
Seasonal correlation	Yes □	No □ Specify			
Cessation	□ Spontaneous	□ after compression	□ by medical intervention		
**REPORT TREATMENT OF THE MOST SEVERE EPISODE**					
Required medical attention?	Yes □	No □			
If yes, please specify:	Consultation only □				
Packing □	# of times		Cauterization □	# of times	
Antifibrinolytics □	# of times		Desmopressin □	# of times	
Replacement therapy □	# of times		Blood transfusion □	# of times	
Notes					

**Cutaneous symptoms** No ☐ If Yes, Trivial ☐ Significant ☐	

AVERAGE PRESENTATION

Type	☐ Petechiae	☐ Bruises	☐ Hematomas
Location of lesions (if any)	☐ Exposed sites	☐ Unexposed sites	☐ Both
Size of average	☐ < 1 cm	☐ 1 – 5 cm	☐ > 5 cm
Minimal or no trauma	Yes ☐	No ☐	

REPORT TREATMENT OF THE MOST SEVERE EPISODE

Required medical attention?	Yes ☐	No ☐
If yes, please specify:	Consultation only ☐	

Notes

Bleeding from minor wounds No ☐ If Yes, Trivial ☐ Significant ☐

AVERAGE PRESENTATION

Number episodes/year	☐ < 1	☐ 1 - 5	☐ 6 - 12	☐ > 12
Duration of average single episode		☐ ≤ 5 mins.	☐ > 5 mins	

REPORT TREATMENT OF THE MOST SEVERE EPISODE

If yes, please specify: Consultation only ☐

Packing ☐	# of times	Cauterization ☐	# of times
Antifibrinolytics ☐	# of times	Desmopressin ☐	# of times
Replacement therapy ☐	# of times	Blood transfusion ☐	# of times

Notes

**Oral cavity bleeding**	No ☐	If Yes,	Trivial ☐ Significant ☐

AVERAGE PRESENTATION

Type of bleeding	☐ Tooth eruption/loss	☐ Gums, spontaneous	☐ Gums, after brushing
	☐ Prolonged bleeding after bites to lip & tongue		☐ Hemorrhagic bullae

REPORT TREATMENT OF THE MOST SEVERE EPISODE

If yes, please specify:	Consultation only ☐		
Packing ☐	# of times	Cauterization ☐	# of times
Antifibrinolytics ☐	# of times	Desmopressin ☐	# of times
Replacement therapy ☐	# of times	Blood transfusion ☐	# of times
Notes			

**Tooth extraction**	No ☐	If Yes,	Trivial ☐ Significant ☐

Total # of teeth extracted ☐☐ # of teeth extracted followed by bleeding ☐☐

MOST SEVERE OCCURRENCE

Age at extraction ☐☐	Type of extraction	☐ Deciduous	☐ Permanent

Prophylaxis before extraction?	☐ None	☐ Antifibrinolytics
	☐ Desmopressin	☐ Replacement therapy
Bleeding after extraction?	Yes ☐	No ☐
Actions taken to control bleeding	☐ None	☐ Consultation only
	☐ Resuturing	☐ Packing
	☐ Antifibrinolytics	☐ Desmopressin
	☐ Replacement therapy	☐ Blood transfusion

Notes

**Gastrointestinal bleeding**	No ☐	Yes ☐

AVERAGE PRESENTATION

# of episodes ☐☐

Type of bleeding ☐ Hematemesis ☐ Melena ☐ Hematochezia

Presence of associated GI disease ☐ Yes ☐ No

☐ Gastritis/ulcer ☐ Colitis ☐ Mallory-Weiss tear

☐ Vascular malformations ☐ Other

REPORT TREATMENT OF THE MOST SEVERE EPISODE

Required medical attention? Yes ☐ No ☐

If yes, please specify: Consultation only ☐ Surgical hemostasis ☐ # of times

Antifibrinolytics ☐ # of times Desmopressin ☐ # of times

Replacement therapy ☐ # of times Blood transfusion ☐ # of times

Notes

**Surgery**	No ☐	If Yes,
Trivial ☐ Significant ☐		

Total # of surgeries ☐☐ # of surgeries followed by bleeding ☐☐

Specify

MOST SEVERE OCCURRENCE

Age at surgery ☐☐ Type of surgery: specify

Prophylaxis before surgery? ☐ None ☐ Antifibrinolytics ☐ Desmopressin ☐ Replacement therapy

Bleeding after surgery? Yes ☐ No ☐

Actions taken to control bleeding ☐ None ☐ Consultation only ☐ Resuturing/surgical

☐ Antifibrinolytics ☐ Desmopressin

☐ Replacement therapy ☐ Blood transfusion

Notes

**Menorrhagia**	N/A ☐	No ☐	Yes ☐

Duration of average menstruation (days) ☐☐ Duration of heavy (days) ☐☐

How often do you change your pads/tampons — on heaviest days ________ hours — on average days ________ hours

What type of feminine product do you use? (i.e. panty liner, super absorbency tampon etc.)

Comments

MOST SEVERE PRESENTATION

Age of maximum severity ☐ 8-12 ☐ 13-16 ☐ 17-20 ☐ >20 yrs

Required medical attention? Yes ☐ No ☐

If yes, please specify:

Pill use ☐ Antifibrinolytics ☐ Dilatation & curettage ☐ # of times

Iron therapy ☐ Desmopressin ☐ Replacement therapy ☐

Blood transfusion ☐ # of times Hysterectomy ☐

Notes

**Post-partum hemorrhage**	N/A ☐	No ☐	If Yes,	Trivial ☐
Significant ☐				

Total # of deliveries ☐☐ # of deliveries followed by bleeding ☐☐

MOST SEVERE OCCURRENCE

Age at delivery ☐☐ Mode of delivery ☐ spontaneous ☐ assisted ☐ c-section

Prophylaxis before delivery ☐ None ☐ Antifibrinolytics ☐ Desmopressin ☐ Replacement therapy

Bleeding after delivery? Yes ☐ No ☐

Actions taken to control bleeding ☐ None ☐ Consultation only

☐ Resuturing/surgical ☐ Antifibrinolytics ☐ Desmopressin

☐ Replacement therapy ☐ Blood transfusion ☐ Hysterectomy

Notes

**Muscle hematomas**	No ☐ Yes ☐

Total # ☐☐

**MOST SEVERE PRESENTATION**					
Please specify type & location					
Post-trauma?		Yes ☐	No ☐		
Prophylaxis?	☐ None	☐ Antifibrinolytics	☐ Desmopressin	☐ Replacement therapy	
Required medical attention?		Yes ☐	No ☐		
If yes, please specify:		Surgical intervention ☐			
		Replacement therapy ☐	Blood transfusion ☐		
Notes					

**Hemarthrosis**	No ☐ Yes ☐

Total # ☐☐

**MOST SEVERE PRESENTATION**					
Please specify type & location					
Post-trauma?		Yes ☐	No ☐		
Prophylaxis?	☐ None	☐ Antifibrinolytics	☐ Desmopressin	☐ Replacement therapy	
Required medical attention?		Yes ☐	No ☐		
If yes, please specify:		Surgical intervention ☐	Desmopressin ☐		
		Replacement therapy ☐	Blood transfusion ☐		
Notes					

**CNS bleeding**	No ☐ Yes ☐

If yes, type of bleeding

Subdural, any intervention	☐	Intracerebral, any intervention	☐

**Other bleeding**	No ☐ Yes ☐

If yes, type of bleeding — Umbilical stump □ Cephalohematoma □

Bleeding at circumcision □ Venipuncture bleeding □

Male, not circumcised □ Male, circumcised □ Female □

Suction Bleeding □ Hematuria, macroscopic

MOST SEVERE PRESENTATION

Please specify type

Required medical attention? Yes □
No □

If yes, please specify:

□ Consultation only **Iron Therapy**

□ Surgical Hemostasis □ Antifibrinolytics

□ Antifibrinolytics □ Desmopressin

□ Replacement therapy □ Blood transfusion

Notes

Other bleeding continued

MOST SEVERE PRESENTATION

Please specify type

Required medical attention? Yes □ No □

If yes, please specify:

□ Consultation only **Iron Therapy**

□ Surgical Hemostasis □ Antifibrinolytics

□ Antifibrinolytics □ Desmopressin

□ Replacement therapy □ Blood transfusion

Notes

Other bleeding continued

MOST SEVERE PRESENTATION

Please specify type

Required medical attention? Yes □ No □

If yes, please specify:

□ Consultation only **Iron Therapy**

- □ Surgical Hemostasis
- □ Antifibrinolytics
- □ Replacement therapy
- □ Antifibrinolytics
- □ Desmopressin
- □ Blood transfusion

Notes

Symptom \ Score	-1	0	1	2	3	4
**Epistaxis**	-	No or trivial (≤5 per year)	>5 per year OR >10 minutes duration	Consultation only	Packing, cauterization or antifibrinolytics	Blood transfusion, replacement therapy or desmopressin
**Cutaneous**	-	No or trivial (≤1cm)	>1cm AND no trauma	Consultation only	-	-
**Minor wounds**	-	No or trivial (≤5 per year)	>5 per year OR >5 minutes duration	Consultation only or Steri-strips	Surgical hemostasis or antifibrinolytics	Blood transfusion, replacement therapy or desmopressin
**Oral cavity**	-	No	Reported at least once	Consultation only	Surgical hemostasis or antifibrinolytics	Blood transfusion, replacement therapy or desmopressin
**Gastrointestinal tract**	-	No	Identified cause	Consultation or spontaneous	Surgical hemostasis, antifibrinolytics, blood transfusion, replacement therapy or desmopressin	-
**Tooth extraction**	No bleeding in at least 2 extractions	None done or no bleeding in 1 extraction	Reported, no consultation	Consultation only	Resuturing, repacking or antifibrinolytics	Blood transfusion, replacement therapy or desmopressin
**Surgery**	No bleeding in at least 2 surgeries	None done or no bleeding in 1	Reported, no consultation	Consultation only	Surgical hemostasis or antifibrinolytics	Blood transfusion, replacement therapy or desmopressin
**Menorrhagia**	-	No	Reported or consultation only	Antifibrinolytics or contraceptive pill use	D&C or iron therapy	Blood transfusion, replacement therapy, desmopressin or hysterectomy
**Post-partum**	No bleeding in at least 2 deliveries	No deliveries or no bleeding in 1 delivery	Reported or consultation only	D&C, iron therapy or antifibrinolytics	Blood transfusion, replacement therapy or desmopressin	-
**Muscle hematoma**	-	Never	Post-trauma, no therapy	Spontaneous, no therapy	Spontaneous or traumatic, requiring replacement therapy or desmopressin	Spontaneous or traumatic, requiring surgical intervention or blood transfusion
**Hemarthrosis**	-	Never	Post-trauma, no therapy	Spontaneous, no therapy	Spontaneous or traumatic, requiring replacement therapy or desmopressin	Spontaneous or traumatic, requiring surgical intervention or blood transfusion
**Central nervous system**	-	Never	-	-	Subdural, any intervention	Intracerebral, any intervention
**Other ***	-	No	Reported	Consultation only	Surgical hemostasis, antifibrinolytics or iron therapy	Blood transfusion, replacement therapy or desmopressin

Table shows the scoring key for the Pediatric Bleeding Questionnaire. In the last row, the symptoms included in the "Other" category are: umbilical stump bleeding, cephalohematoma, post-circumcision bleeding, post-venipuncture bleeding, and macroscopic hematuria.
